# Evaluation of the use of raw glycerol in biomass production by *Trichoderma reesei *QM9414

**DOI:** 10.1186/1753-6561-8-S4-P173

**Published:** 2014-10-01

**Authors:** Kally Alves de Sousa, Genilton da Silva Faheina Junior, Karine Thiers Leitão Lima, Gustavo Adolfo Saavedra Pinto, Rilvia Saraiva de Santiago Aguiar, Diana Cristina da Silva Azevedo

**Affiliations:** 1 Federal University of Ceará, Department of Chemical Engineering, Fortaleza, CE, Brazil; 2 Federal University of Ceará, Department of Biochemistry and Molecular Biology, Fortaleza, CE, Brazil; 3 Brazilian Agricultural Research Corporation (Embrapa), National Research Center of Tropical Agroindustry, Fortaleza, CE, Brazil

## Background

Biodiesel has emerged as a energy, environmental and social solution proposal. However, there are some technical challenges that need to be solved, for example, use of the by-product of biodiesel production, crude glycerol (CG). To avoid future problems caused by the accumulation of CG and simultaneously make biodiesel production more competitive, the search for the most viable alternatives to the use of this waste has been proposed. The CG can be the raw material for the synthesis of products with high added value, obtained through biochemical conversion (fermentation routes) [1-3]. The biomass of *Trichoderma *is considered an excellent biological control agent for pest and disease pathogens in plants and is promising alternative to conventional chemical control [4,5]. Thus, the aim of this study was to preliminarily evaluate the use of CG in biomass production of *Trichoderma reesei *QM9414 by submerged fermentation.

## Methods

The fermentations were conducted in flasks of 250 ml capacity (100 ml of culture broth). The inoculum represented about 1% (v.v^-1^) from the fermentation medium. The incubation time was 72 hours at 30 ° C under agitation at 175 rpm. The initial pH of the tests was 5.5. The basic medium of fermentation (test T1) presented in g.L^-1^: 15.0 of CG, 1.4 of (NH_4_)_2_SO_4_, 2.0 of KH_2_PO_4_, 0.3 of CaCl_2_.2H_2_O, 0.6 of MgSO_4_.7H_2_O, 0.005 of FeSO_4_.7H_2_O, 0.002 of CoCl_2_.6H_2_O; 0.0016 of MnSO_4_.H_2_O; 0.0014 of ZnSO_4_.7H_2_O and 6.0 yeast extract (YE). It was also tested modifications of the basic medium: YE suppression (test T2); YE suppression and increased (NH_4_)_2_SO_4 _(2.8 g.L^-1^) (test T3); decreased YE (0.6 g.L^-1^) (test T4); decreased YE (0.6 g.L^-1^) and increased (NH_4_)_2_SO_4 _(2.8 g.L^-1^) (test T5). After 72 hours, was analyzed the consumption of glycerol (high performance liquid chromatography) and the produced biomass (dry weight).

## Results and conclusions

The CG used in the study came from the production of biodiesel from soybeans (purity of 80.9%). It was found biomass of *T. reesei *QM9414 in all fermentation tests using CG. However, the presence of YE (6.0 g.L^-1^) in higher concentration in the fermentation medium (T1) provided the best results for the production of biomass (4.49 g.L^-1^). The lower production of biomass, 1.32 and 1.52 g.L^-1^, were detected, respectively, in tests T2 and T3. It was noticed that the isolated presence of inorganic nitrogen source ((NH_4_)_2_SO_4_), even at a concentration of 2.8 g.L^-1^, did not ensure an increase in biomass production. Tests T4 and T5 were respectively 2.33 and 2.48 g.L^-1 ^of biomass. Regarding the consumption of glycerol, tests conducted with YE (T1, T4 and T5) were related to higher percentage (over 73.0%). The lowest percentages of glycerol consumption occurred in the tests that employed exclusively (NH_4_)_2_SO_4 _(T2 and T3). The results showed that the presence of yeast extract favored the use of crude glycerol and allowed the formation of biomass. It was verified the viability of using crude glycerol as carbon source for the production of biomass *T. reesei *QM9414.
